# Equipment for neonatal resuscitation in a middle-income country: a national survey in Vietnam

**DOI:** 10.1186/s12887-016-0664-0

**Published:** 2016-08-20

**Authors:** Daniele Trevisanuto, Francesco Cavallin, Gaston Arnolda, Tran Dinh Chien, Ornella Lincetto, Ngo Minh Xuan, Nguyen Viet Tien, Nguyen Thi Xuan Hoi, Luciano Moccia

**Affiliations:** 1 Women’s and Children’s Health Department, Medical School, University of Padua, Azienda Ospedaliera di Padova, Via Giustiniani, 3, 35128 Padua, Italy; 2 Amici della Neonatologia Trentina (ANT), Trento, Italy; 3 Independent statistician, Padua, Italy; 4 Thrive Networks, Oakland, CA USA; 5 School of Public Health & Community Medicine, Faculty of Medicine, University of New South Wales, Sydney, NSW Australia; 6 World Health Organization, Country Office Bhutan, Thimphu, Bhutan; 7 University of Medicine Pham Ngoc Thach, Ho Chi Minh City, Vietnam; 8 Perinatal and Neonatal Association of Ho Chi Minh City, Ho Chi Minh City, Vietnam; 9 National Hospital of Obstetrics and Gynecology, Hanoi, Vietnam

**Keywords:** Equipment, Middle-income country, Neonatal resuscitation, Survey, Vietnam

## Abstract

**Background:**

Interventions to improve neonatal resuscitation are considered a priority for reducing neonatal mortality. In addition to training programs for health caregivers, the availability of adequate equipment in all delivery settings is crucial. In this study, we assessed the availability of equipment for neonatal resuscitation in a large sample of delivery rooms in Vietnam, exploring regional differences.

**Methods:**

In 2012, a structured questionnaire on 2011 neonatal resuscitation practice was sent to the heads of 187 health facilities, representing the three levels of hospital-based maternity services in eight administrative regions in Vietnam, allowing national and regional estimates to be calculated.

**Results:**

Overall the response rate was an 85.7 % (160/187 hospitals). There was a limited availability of equipment considered as “essential” in the surveyed centres: stethoscopes (68.0 %; 95 % CI: 60.3–75.7), clock (50.3 %; 42.0–58.7), clothes (29.5 %; (22.0–36.9), head covering (12.3 %; 7.2–17.4). The percentage of centres equipped with polyethylene bags (2.2 %; 0.0–4.6), pulse oximeter (9.4 %; 5.2–13.6) and room air source (1.9 %; 0.1–3.6) was very low.

**Conclusion:**

Adequate equipment for neonatal resuscitation was not available in a considerable proportion of hospitals in Vietnam. This problem was more relevant in some regions. The assessment strategy used in this study could be useful for organizing the procurement and distribution of supplies and equipment in other low and/or middle resource settings.

## Background

Worldwide each year about 6.6 million children under 5 years of age die and of these 44 % are newborns. Intrapartum-related events, previously called “birth asphyxia”, account for a quarter of neonatal deaths and almost all of them (99 %) occur in low-middle income countries [1].

Interventions to improve neonatal resuscitation are an essential part of any strategy to reduce neonatal mortality. Basic neonatal resuscitation and care in the so-called golden minute after birth may decrease neonatal mortality in low-resource settings and it is estimated to reduce full-term infant deaths by up to 30 % [2]. In addition to training programs for providers involved in the management of the neonates at birth, the availability of adequate equipment in all delivery settings is crucial. A recent review of data from 6 African countries showed that the percentage of birthing facilities with equipment for neonatal respiratory support ranged from 8 to 22 % [2]. Simplified algorithms and lists of essential equipment for neonatal resuscitation at first referral level and higher in low-resource settings are recommended by international institutions such as World Health Organization (WHO) and American Academy of Pediatrics (AAP) [3, 4]. According to the WHO and the AAP “Helping Babies Breathe” program the list of essential equipment should include gloves, towels/cloths, head covering, scissors, ties, suction device, ventilation device, stethoscope and timer [4].

South-East Asia is the regional area with the highest proportion of under-five mortality attributable to neonatal causes [5]. As Vietnam has implemented many initiatives and humanitarian plans in recent years [6], it is widely seen as a model among middle-income countries for the significant improvements in economy and health status. However, important challenges, such as the reduction of preventable neonatal mortality [5–7], and the disparity between different geographical and socio-economic regions [8–12], remain to be addressed.

In a previous study, we evaluated consistency of resuscitation practices and adherence to the international guidelines for neonatal resuscitation in a large representative sample of hospitals in Vietnam [13]. The present study reports data regarding the equipment available for neonatal resuscitation in the same sample of Vietnamese delivery rooms (DR), exploring regional differences.

## Methods

### Participants and evaluation instrument

In 2012, we conducted a survey of DR management among hospital based-obstetric and neonatal services in Vietnam [13]. The survey was performed using a structured questionnaire regarding DR practices in the domains of neonatal resuscitation (first part) and the equipment available at the centre (second part). (Additional File 1) The questions included multiple choices, brief responses, and yes/no questions. The questions all referred to the period 1 January to 31 December 2011.

**Fig. 1:**
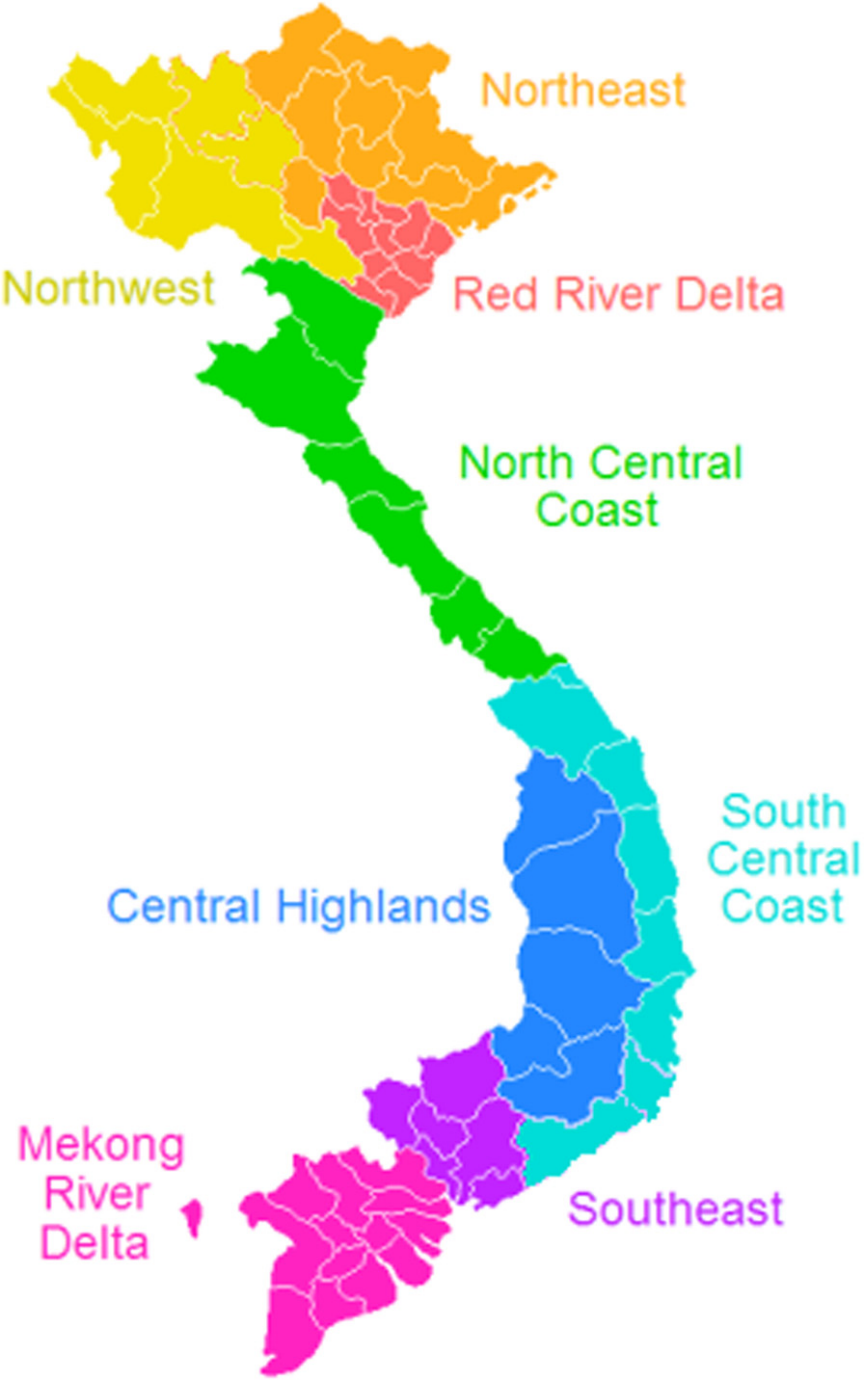
Administrative regions in Vietnam. (source: CC BY-SA 3.0, https://commons.wikimedia.org/w/index.php?curid=185025)

Details of the survey have been previously published [13]. Briefly, 187 hospitals were chosen among the 610 hospitals with ≥500 births in 2010 by (i) a census of all 6 central hospitals and all 72 provincial hospitals and (ii) a 20 % sample survey of 532 district hospitals (*n* = 109) with ≥500 births in 2011. The district hospitals were chosen from within each of the eight administrative regions commonly used for reporting purposes by the Government of Vietnam (Fig. 1), with each region restricted to providing 20 % of the district hospitals in its catchment, following a stratified random sample with proportional allocation approach.

In this article, we report data referred to the second part (equipment) of the questionnaire. We considered the equipment as a) essential (included in the list of HBB algorithm) and b) advanced (recommended by the 2010 International Liaison Committee on Resuscitation (ILCOR) guidance and by the American Heart Association (AHA) [14, 15].

### Statistics

The design of the sample survey has been described elsewhere [13]. In the present paper, all estimates are the weighted aggregate of central, provincial and district level estimates after reweighting for sampling fraction, for district hospitals only, and nonresponse, for all three levels. No survey respondents failed to answer questions regarding equipment, therefore no additional reweighting for missing data was needed. National estimates and regional estimates (with 95 % Confidence Intervals) of available equipment were weighted to account for 100 % sampling of central and provincial hospitals versus approximately 20 % sampling of districts, and the exact sampling fractions were used for weighting. As the response rate was differential, regional data were also inflated by the inverse of the local response rate to ensure no systematic distortion of the estimated group parameters. Statistical analysis was performed using SAS v9.2 (SAS Institute Inc., Cary, NC, USA).

## Results

The number of participants according to hospital level and administrative region is shown in Table 1. Overall, the response rate for the equipment section of the questionnaire was 85.7 % (160/187 hospitals). The response rate was 84.4 % (27/32) in Red River Delta, 88.0 % (22/25) in North Central Coast, 86.7 % (26/30) in Northeast, 100 % (10/10) in Northwest, 80.0 % (12/15) in Central Highlands, 72.7 % (16/22) in South Central Coast, 94.4 % (17/18) in Southeast and 85.7 % (30/35) in Mekong River Delta. The estimates of equipment availability in each of the eight regions are reported in Table 2 (essential equipment) and Table 3 (advanced equipment).

**Table 1:** Number of participants according to hospital level and administrative region

		Central	Provincial	District	Total
Northern VN	Red	1	12	14	27
	North central	2	5	15	22
	North east	1	13	12	26
	North west	0	4	6	10
Southern VN	Central highlands	0	6	8	12
	South central	0	7	9	16
	South eastern	1	5	11	17
	Mekong	1	13	16	30
	Total	6	63	91	160

**Table 2:** Essential equipment

ESTIMATES		Northern VN				Southern VN			
	Overall (n. 160)	Red (n. 27)	North central (n. 22)	North east (n. 26)	North west (n. 10)	Central highlands (n. 12)	South central (n. 16)	South eastern (n. 17)	Mekong (n. 30)
Scissors	99.0 (97.7–100.0)	98.8 (97.9–99.7)	100.0 (100.0–100.0)	100.0 (100.0–100.0)	85.4 (61.3–100.0)	100.0 (100.0–100.0)	100.0 (100.0–100.0)	100.0 (100.0–100.0)	100.0 (100.0–100.0)
Suction device	97.2 (94.5–99.9)	100.0 (100.0–100.0)	100.0 (100.0–100.0)	100.0 (100.0–100.0)	70.8 (40.2–100.0)	100.0 (100.0–100.0)	97.9 (96.1–99.7)	100.0 (100.0–100.0)	94.5 (84.9–100.0)
Gloves	95.1 (91.6–98.6)	92.8 (82.2–100.0)	92.6 (81.9–100.0)	98.8 (97.8–99.8)	100.0 (100.0–100.0)	100.0 (100.0–100.0)	88.4 (72.2–100.0)	98.5 (97.2–99.7)	94.5 (84.9–100.0)
Ties	89.5 (84.5–94.5)	85.5 (71.2–99.9)	79.1 (62.1–96.2)	97.6 (96.1–99.1)	70.8 (40.3–100.0)	89.0 (70.3–100.0)	97.9 (96.1–99.7)	100.0 (100.0–100.0)	89.0 (75.9–100.0)
Face mask	84.2 (78.0–90.3)	84.3 (69.9–98.7)	81.8 (64.7–98.8)	71.0 (50.4–91.6)	50.0 (17.6–82.4)	89.0 (70.3–100.0)	100.0 (100.0–100.0)	83.5 (64.3–100.0)	94.5 (84.9–100.0)
Stethoscope	68.0 (60.3–75.7)	75.9 (59.0–92.7)	82.7 (68.1–97.3)	90.7 (78.6–100.0)	53.1 (20.7–85.6)	25.0 (0.5–49.5)	57.9 (32.3–83.5)	66.9 (43.0–90.8)	61.8 (42.6–81.1)
Clock	50.3 (42.0–58.7)	59.2 (39.4–79.0)	41.8 (20.9–62.7)	56.9 (35.4–78.4)	20.8 (0.0–45.2)	22.1 (0.0–46.6)	46.3 (20.7–72.0)	57.1 (32.4–81.9)	63.6 (44.3–82.4)
Self-inflating bag	49.2 (40.9–57.5)	58.9 (38.6–79.3)	27.9 (9.0–46.7)	47.8 (26.2–69.4)	3.1 (0.2–6.0)	47.1 (18.7–75.4)	69.5 (45.1–93.8)	33.9 (11.5–56.2)	69.1 (50.6–87.5)
Flow-inflating bag	49.4 (41.1–57.7)	44.6 (24.3–65.0)	52.6 (31.3–73.8)	53.3 (31.7–74.9)	38.5 (7.8–69.3)	64.0 (36.6–81.4)	36.8 (12.4–61.2)	70.7 (48.5–92.8)	39.9 (20.6–59.2)
Ventilatory device (self-inflating bag or flow-inflating bag)	82.7 (76.3–89.1)	86.7 (72.4–100.0)	74.4 (55.5–93.2)	83.2 (67.4–99.9)	41.7 (10.7–72.7)	77.9 (53.4–100.0)	81.0 (59.6–100.0)	100.0 (77.4–100.0)	91.7 (84.9–100.0)
Towels/Cloths	29.5 (22.0–36.9)	43.3 (23.0–63.6)	32.6 (12.5–52.7)	19.8 (3.5–36.1)	0	8.8 (3.1–14.6)	15.8 (0.0–32.2)	36.1 (12.2–60.0)	44.5 (24.8–64.2)
Head covering	12.3 (7.2–17.4)	22.9 (6.0–39.8)	0	6.0 (3.0–9.0)	0	2.9 (0.4–5.5)	15.8 (0.0–32.2)	18.0 (0.0–37.2)	19.9 (4.4–35.5)

Data expressed as rate estimates (95 % CI). No missing answers

**Table 3:** Advanced equipment

ESTIMATES		Northern VN				Southern VN			
	Overall (n. 160)	Red (n. 27)	North central (n. 22)	North east (n. 26)	North west (n. 10)	Central highlands (n. 12)	South central (n. 16)	South eastern (n. 17)	Mekong (n. 30)
Oxygen source	100.0 (100.0–100.0)	100.0 (100.0–100.0)	100.0 (100.0–100.0)	100.0 (100.0–100.0)	100.0 (100.0–100.0)	100.0 (100.0–100.0)	100.0 (100.0–100.0)	100.0 (100.0–100.0)	100.0 (100.0–100.0)
Laryngoscope	62.8 (54.6–70.9)	16.6 (5.3–27.9)	59.6 (38.7–80.4)	40.9 (20.1–61.6)	20.8 (0.0–45.2)	89.0 (70.3–100.0)	71.6 (47.2–95.9)	91.7 (77.5–100.0)	100.0 (100.0–100.0)
Endotracheal tubes	58.5 (50.2–66.7)	28.7 (11.6–45.8)	46.1 (24.8–67.3)	42.1 (21.2–62.9)	35.4 (4.9–66.0)	66.9 (39.5–94.4)	69.5 (45.1–93.8)	83.5 (64.3–100.0)	88.2 (75.0–100.0)
Infant warmer	53.4 (45.1–61.7)	33.6 (14.9–52.2)	62.2 (41.3–83.1)	33.9 (14.7–53.2)	82.3 (58.1–100.0)	77.9 (53.4–100.0)	48.4 (22.7–74.1)	58.7 (33.9–83.5)	59.8 (40.1–79.6)
Reservoir for ambu bag	53.1 (44.8–61.4)	43.4 (23.1–63.8)	35.3 (15.2–55.4)	15.2 (2.8–27.7)	17.7 (0.0–41.9)	75.0 (50.5–99.5)	78.9 (57.6–100.0)	68.7 (33.9–83.5)	86.4 (73.3–99.6)
Pulse oximeter	9.4 (5.2–13.6)	8.4 (0.0–19.0)	1.3 (0.3–2.9)	21.0 (4.6–37.4)	3.1 (0.2–6.0)	11.0 (0.0–29.7)	2.1 (0.3–3.9)	12.9 (0.0–27.2)	10.7 (0.9–20.5)
Ventilator	6.1 (2.3–9.8)	7.2 (0.0–17.8)	1.1 (0.7–1.5)	2.3 (1.1–3.6)	14.6 (0.0–38.7)	0	9.5 (0.0–25.7)	11.3 (0.0–25.6)	7.2 (0.0–16.9)
Laryngeal mask airway	2.7 (0.2–5.3)	1.2 (0.3–2.1)	0	0	3.1 (0.2–6.0)	14.0 (0.0–32.7)	9.5 (0.0–25.7)	1.5 (0.8–2.2)	0
Polyethylene bags	2.2 (0.0–4.6)	7.2 (0.0–17.8)	0	1.2 (0.2–2.2)	0	0	0	8.3 (0.0–22.5)	0
Room air source	1.9 (0.1–3.6)	7.2 (0.0–17.8)	0	0	6.3 (01.4–11.1)	0	2.1 (0.3–3.9)	1.5 (0.8–2.2)	0
Blood gas analyzer	1.2 (0.0–3.1)	0	0	1.1 (0.7–1.6)	0	0	0	0	5.5 (0.0–15.1)

Data expressed as rate estimates (95 % C.I.)

Midwives were more frequently responsible for equipment preparation and check (Table 4). Overall, the availability of a written checklist was limited (Table 5).

**Table 4:** Health caregiver responsible for equipment check

ESTIMATES		Northern VN				Southern VN			
	Overall (n. 160)	Red (n. 27)	North central (n. 22)	North east (n. 26)	North west (n. 10)	Central highlands (n. 12)	South central (n. 16)	South eastern (n. 17)	Mekong (n. 30)
Midwife	83.2 (76.9–89.4)	84.3 (69.9–98.7)	74.4 (55.5–93.2)	83.7 (67.5–100.0)	70.8 (40.2–100.0)	77.9 (53.4–100.0)	78.9 (57.5–100.0)	97.0 (94.6–99.0)	89.0 (75.9–100.0)
Physician	18.5 (12.0–25.1)	20.5 (3.7–37.4)	24.3 (5.5–43.1)	31.4 (10.8–51.9)	17.7 (0.0–41.9)	11.0 (0.0–29.7)	19.0 (0.0–40.4)	1.5 (0.3–2.8)	15.3 (2.1–28.5)
Nurse	8.6 (4.1–13.0)	1.2 (0.3–2.2)	13.5 (0.0–28.0)	15.1 (0.0–31.3)	0	27.9 (3.4–52.5)	13.7 (0.0–30.0)	3.0 (1.0–5.0)	0

Data expressed as rate estimates (95 % C.I.)

**Table 5:** Availability of written checklist

ESTIMATES		Northern VN				Southern VN			
	Overall (n. 160)	Red (n. 27)	North central (n. 22)	North east (n. 26)	North west (n. 10)	Central highlands (n. 16)	South central (n. 16)	South eastern (n. 17)	Mekong (n. 30)
Availability of written checklist	39.4 (31.3–47.4)	59.2 (39.4–79.0)	28.1 (9.2–46.9)	20.0 (6.9–33.1)	6.3 (1.4–11.1)	47.1 (18.7–75.4)	44.2 (18.6–69.8)	30.9 (8.7–53.0)	54.3 (34.4–74.2)

Data expressed as rate estimates (95 % C.I.)

## Discussion

In this study, we assessed the equipment available for neonatal resuscitation in a large sample of Vietnamese delivery rooms, exploring regional differences. Our results show that a large proportion of the surveyed hospitals lack equipment classified as “essential” in the WHO and HBB algorithm. This problem was more marked in some regions than others. These data could be useful for guiding a program to supply and distribute material and equipment, to ensure that all birthing facilities in the country have the essential supplies.

In Vietnam, some of the essential equipment for neonatal resuscitation, such as scissors, suction devices, gloves, ties, ventilatory devices including self-inflating bag and flow-inflating bag, and face-masks was available in the majority of the hospitals. The availability of other relevant elements (stethoscope, clock, clothes and head covering) included in the WHO and HBB list were clearly insufficient. The lack of equipment was more marked in some administrative regions such as the Central Highlands and North west areas.

2010 AHA Guidelines for Neonatal Resuscitation recommend that heart rate should be detected with a stethoscope or electrocardiography because umbilical cord palpation is less accurate [14, 15]. Time registration is an important activity during neonatal resuscitation but, surprisingly, about half of the hospitals declared that a clock was not available in their delivery room [14–16]. Finally, prevention of thermal losses at birth is a crucial goal to reduce mortality and morbidity in neonates, especially preterm infants [17]. A combination of interventions such as environmental temperature at 23-25 °C, infant warmers, plastic bags, pre-warmed blankets and head covering is recommended to avoid hypothermia [14–16, 18, 19]. Notably, only 12.3 % of hospitals reported the availability of a head covering for preventing neonatal hypothermia in delivery room while only 2.2 % reported having a plastic bag. The effectiveness of a plastic bag on prevention of postnatal hypothermia in preterm infants has been demonstrated in high as well as low resource settings [20–23]. Implementation of these low-cost interventions in delivery room could significantly improve neonatal outcomes.

Turning to the list of “advanced” equipment, it is notable that only 9.4 % of the Vietnamese hospitals have a pulse oximeter in the delivery room. 2010 Guidelines for Neonatal Resuscitation state that “It is recommended that oximetry be used when resuscitation can be anticipated, when positive pressure is administered for more than a few breaths, when cyanosis is persistent, or when supplementary oxygen is administered” [15]. The inability to titrate oxygen supplementation guided by an oximeter may expose neonates to high oxygen concentrations and, consequently, increase mortality [24]. In addition, the availability of a room air source to allow blending was very limited (1.9 % of the sample); this could be a serious problem because the initial oxygen concentrations for preterm infants (<35 weeks gestation) should be 21–30 % [16]. In these circumstances, a scheme correlating the oxygen flow rate and the corresponding delivered oxygen concentrations when using a neonatal self inflating bag can be used [25, 26]. Although the availability of written checklists for equipment and material preparation is an important organizational issue [14–16], they were routinely used in only 39.4 % of surveyed centers. This is another important aspect that needs to be improved.

Similar national or local surveys have been previously conducted in high [27–29] as well in low resource settings [30, 31]. These studies aimed to assess the practices of health caregivers and their adherence to official guidelines more than the availability of equipment [27–29]. Also these studies showed marked variation among the hospitals and/or the regional areas within the same country [13, 27–31].

A recent study examined progress in the implementation of the basic emergency obstetric and neonatal care (EmONC) in Addis Ababa comparing two periods: 2008 (before the intervention) and 2013 (after the intervention) [31]. The results show that there were advances in infrastructure, medical supplies and personnel for EmONC provision. However, providers knowledge scores on diagnosis and management of labor, bleeding after childbirth, birth asphyxia and skill scores on neonatal resuscitation did not improved between the two periods suggesting that, in addition to infrastructure and medical equipment, staff education remains a key point for improving the quality of maternal and neonatal care [27–31].

As the relationship between the quality of health providers’ practice and the availability of adequate equipment has been previously demonstrated [1, 32], the results of this study should be interpreted together with the data obtained from our previous survey [13]. This approach will help to obtain an objective picture of the regional areas that need more educational and technical investments.

The strength of this study is national representation and the high response rate (85.6 %) of the enrolled hospitals, with good representation of all administrative regions. However, there are some limitations to this study. We involved only the directors of the participating centres and relied on self-report, without inspection of the sites and observation of clinical practices. The sampling strategy give us confidence in the accuracy of the national estimates, but the response rate, while high at 85.7 % overall, leaves open the possibility of selection biases if responding hospitals have higher or lower rates of equipment availability than non-respondents. As the survey was conducted in 2012 and based on 2011 practice, changes may have already occurred during the last 5 years.

## Conclusion

Our study reports on the equipment available for neonatal resuscitation in a large representative sample of Vietnamese hospitals. Our results show that the equipment classified as “essential” by the WHO guidelines and HBB algorithm was not available in a considerable portion of the surveyed hospitals. This problem was more relevant in some regions. Our data could provide a basis for ensuring that all Vietnamese hospitals that offer maternity services have a full complement of essential and advanced resuscitation equipment. The assessment strategy used in this study could be replicated as a basis for organizing the supply and distribution of material and equipment in other low and/or middle resource settings.

## Abbreviations

AAP, American Academy of Pediatrics; AHA, American Heart Association; DR; delivery room; HBB, helping babies breathe; WHO, World Health Organization
